# Next Generation Sequencing for Detection and Discovery of Plant Viruses and Viroids: Comparison of Two Approaches

**DOI:** 10.3389/fmicb.2017.01998

**Published:** 2017-10-13

**Authors:** Anja Pecman, Denis Kutnjak, Ion Gutiérrez-Aguirre, Ian Adams, Adrian Fox, Neil Boonham, Maja Ravnikar

**Affiliations:** ^1^Department of Biotechnology and Systems Biology, National Institute of Biology, Ljubljana, Slovenia; ^2^Jožef Stefan International Postgraduate School, Ljubljana, Slovenia; ^3^Fera Science Ltd., York, United Kingdom; ^4^Institute for Agri-Food Research and Innovation, Newcastle University, Newcastle upon Tyne, United Kingdom

**Keywords:** next generation sequencing, small RNA, ribosomal RNA depleted total RNA, detection, plant viruses, plant viroids

## Abstract

Next generation sequencing (NGS) technologies are becoming routinely employed in different fields of virus research. Different sequencing platforms and sample preparation approaches, in the laboratories worldwide, contributed to a revolution in detection and discovery of plant viruses and viroids. In this work, we are presenting the comparison of two RNA sequence inputs (small RNAs vs. ribosomal RNA depleted total RNA) for the detection of plant viruses by Illumina sequencing. This comparison includes several viruses, which differ in genome organization and viroids from both known families. The results demonstrate the ability for detection and identification of a wide array of known plant viruses/viroids in the tested samples by both approaches. In general, yield of viral sequences was dependent on viral genome organization and the amount of viral reads in the data. A putative novel *Cytorhabdovirus*, discovered in this study, was only detected by analysing the data generated from ribosomal RNA depleted total RNA and not from the small RNA dataset, due to the low number of short reads in the latter. On the other hand, for the viruses/viroids under study, the results showed higher yields of viral sequences in small RNA pool for viroids and viruses with no RNA replicative intermediates (single stranded DNA viruses).

## Introduction

Plant viruses and viroids are important plant pathogens, causing economic losses by reducing crop quality and quantity all over the world (Loebenstein, 2008; Soliman et al., 2012). Thus, their reliable detection is of a crucial importance for plant protection. Classical methods in plant virus diagnostics can be roughly divided into specific (serological/molecular tests) and non-specific (indicator test plants, electron microscopy) approaches. Specific methods are usually targeted to one or a few viral species and require *a priori* knowledge of the pathogens being tested, whilst non-specific approaches do not require specific knowledge of the pathogens, however, frequently only classify viruses at a genus level based on the shared physical/biological characters. Discovery of new viruses/viroids and new hosts has increased rapidly after the introduction of next generation sequencing (NGS). NGS technologies allow a generic approach (non-specific method) to virus identification that does not require any prior knowledge on the targeted pathogens but can deliver a species/strain specific result (Adams and Fox, 2016). It was first employed for plant virus detection in 2009 (Adams et al., 2009; Al Rwahnih et al., 2009; Kreuze et al., 2009). Since 2009, different sample preparation methods have been developed, relying on different nucleic acid inputs, most commonly: total RNA (totRNA); ribosomal RNA depleted total RNA (rRNA depleted totRNA); double stranded RNA (dsRNA); virus derived small interfering RNA (sRNA); RNA from purified or partially purified viral particles; polyadenylated RNA (poly(A) RNA); and RNA after subtractive hybridization with healthy plant RNA. Applications of different sample preparation methods are reviewed in Roossinck et al. (2015); Wu et al. (2015), and Adams and Fox (2016). Viruses have diverse genome organizations and use different replication strategies. Based on these two characteristics they can be classified into 7 groups (the Baltimore classification): double stranded DNA (Group I, dsDNA +/−), single stranded DNA (Group II, ssDNA +), double stranded RNA (Group III, dsRNA +/−), positive sense single stranded RNA (Group IV, ssRNA +), negative sense single stranded RNA (Group V, ssRNA −) viruses, positive sense single stranded RNA viruses that replicate through a DNA intermediate (Group VI, ssRNA-RT +), and double stranded DNA viruses that replicate through a RNA intermediate (Group VII, dsDNA-RT +/−) (Baltimore, 1971). Viroids are classified into two families: members of *Avsunviroidae* family replicate in chloroplast, whereas members of *Pospiviroidae* family replicate in nucleus (Flores et al., 2014). Considering the diversity of viruses and viroids, with different genome organizations in mind, it is conceivable that using different nucleic acid inputs for NGS could affect their overall detection.

Sample preparation methods (i.e., different nucleic acid inputs), used before NGS, can differ in their efficiency and can have specific advantages and disadvantages. For example, subtractive hybridization of the host plant nucleic acids, using tomato (*Solanum lycopersicum*) and *Pepino mosaic virus* (PepMV, RNA +, *Potexvirus, Alphaflexiviridae*) as a model system, resulted in three times more PepMV sequences in subtracted sample (Adams et al., 2009), but as it is a time consuming procedure, which requires a healthy plant of the same species as the sample to be tested (Adams and Fox, 2016), subtractive hybridization is not well suited in a high-throughput diagnostic settings. Some sample preparation methods may cause bias in the detection of a particular group of viruses. Sequencing of dsRNA was mainly used for detection of RNA + and RNA +/− viruses, since RNA—and DNA viruses could be missed (Roossinck et al., 2015) using this approach; nevertheless, a new geminivirus (DNA +) was identified using dsRNA sequencing (Al Rwahnih et al., 2013). RNA isolated from purified viral particles has been successfully used for sequencing different viruses (reviewed in Roossinck et al., 2015; Wu et al., 2015). A comparison between deep sequencing of sRNAs and RNA isolated from viral particles showed higher efficiency of the latter for the reconstruction of complete consensus *Potato virus Y* (RNA +, *Potyvirus, Potyviridae*) genomes (Kutnjak et al., 2015). However, virus purification is not applicable for un-encapsidated viruses and requires sample specific processing since it is unlikely that all viruses could be captured by a single protocol for viral particles purification (Roossinck et al., 2015; Wu et al., 2015). Poly(A) RNA based enrichment strategy has been also used for both RNA and DNA viruses but it is not applicable for the detection of viruses without a poly(A) tail (Wu et al., 2015). Data from sequencing poly(A) RNA showed a lower degree of virus genome coverage in comparison to saturated genome coverage reached with sRNA data for *Grapevine leafroll-associated virus 3* (RNA +, *Ampelovirus, Closteroviridae*), yet a comparison between poly(a)RNA and sRNA data for *Hop stunt viroid*, (*Pospiviroidae* family) showed comparable outcomes (high genome coverage) for both approaches (Visser et al., 2016).

In this study, we focused the comparison (with the detection and identification of plant viruses and viroids in mind) on the two types of RNA inputs: sequencing of sRNA and sequencing of rRNA depleted totRNA. Those two approaches seem to be the most generically applicable to viruses with different genome types and replication strategies and could be relatively easily integrated in workflows of diagnostic labs.

Sequencing and assembly of viral sRNA (Kreuze et al., 2009) has been successfully used for detection and identification of several plant viruses and viroids and their complete genome assembly (reviewed in Boonham et al., 2014; Kreuze, 2014). It has been speculated that this approach could be problematic if used to detect viruses that either do not trigger silencing responses or that express silencing suppressors (Roossinck et al., 2015). Also, de novo assembly of longer viral contigs could be complicated due to short reads lengths (Boonham et al., 2014; Roossinck et al., 2015; Adams and Fox, 2016). On the other hand, the approach is very generic, using the same protocol of sample preparation for many different plant species and doesn't require high quality of RNA input (Kutnjak et al., 2017).

Sequencing of plant viruses using total RNA as an input was first described by Adams et al. (2009) and Al Rwahnih et al. (2009), followed by several successful studies (reviewed in Boonham et al., 2014). It is also a very generic approach, however, a potential shortcoming of that method can be the low viral RNA titer within the background plant RNA. To overcome this, removal of the highly abundant plant ribosomal RNA from the total RNA pool (rRNA depleted tot RNA) has been explored, which can results in a 10-fold enrichment of viral RNA (Adams and Fox, 2016).

Recent comparison (Visser et al., 2016) of sRNA and rRNA depleted totRNA for *Citrus tristeza virus* (RNA +, *Closterovirus, Closteroviridae*) and *Citrus dwarfing viroid* (*Pospiviroidae* family) implied a preferential use of rRNA depleted totRNA for *de novo* assembly of viral genome sequences from NGS data. No wider comparison of these two approaches (including viruses with different genome characteristics) has been reported. With this in mind, our aim was to compare the two approaches, including plant viruses with different genome structures and replication strategies (belonging to different Baltimore classification groups) and viroids from different families into comparison. The aims were to compare the two approaches in terms of: (1) known virus detection and identification (2) recovery of virus/viroid reads and (3) effectiveness of detection of new/unknown viruses by reconstruction of longer viral contigs by *de novo* assembly and read mapping analysis approaches.

## Materials and methods

### Description of samples

Nine virus-infected plant samples were included in this study. The selection included samples of different plant species, infected with a range of plant viruses in single or mixed infections with at least one representative from each group of the Baltimore viral classification containing plant viruses, and viroids from both families (Table 1).

**Table 1:** Samples included in the comparison with corresponding results from: NGS (viruses/viroids listed in the table were detected in corresponding samples by NGS) and other diagnostic methods (ELISA, RT-PCR and RT-qPCR).

**Sample number**	**Virus, genus, family**	**Baltimore classification**	**Genome organization**	**Abbreviations**	**Host**	**Initial detection with NGS**		**Results of confirmatory testing**	**NCBI GenBank accession number**	**NCBI SRA accession number (sRNA/rRNA depleted totRNA)**
						**sRNA**	**rRNA depleted totRNA**			
I	^*^Potato virus Y, Potyvirus, Potyviridae	Group IV (ssRNA +)	Linear	PVY	*Solanum tuberosum*	+	+	+^a^	KY810782	SRR5377154/SRR5377146
II	^*^Cauliflower mosaic virus, Caulimovirus, Caulimoviridae;	Group VII (dsDNA-RT +/–)	Circular	CaMV	*Brassica oleracea*	+	+	+^a^	KY810770	SRR5377153/SRR5377145
	Novel cabbage cytorhabdovirus 1, *Cytorhabdovirus, Rhabdoviridae*	Group V (ssRNA –)	Linear	Novel CCyV1	*Brassica oleracea*	–	+	+^b^	KY810772	
III	^*^Tomato Yellow Leaf Curl Virus, Begomovirus, Geminiviridae;	Group II (ssDNA +)	Circular	TYLCV	*Solanum lycopersicum*	+	+	+^a^	KY810789	SRR5377152/SRR5377144
	*Tomato chlorosis virus, Crinivirus, Closteroviridae;*	Group IV (ssRNA +)	Linear, segmented	ToCV	*Solanum lycopersicum*	+	+	+^b^	KY810786 KY810787	
	*Pepino mosaic virus, Potexvirus, Alphaflexiviridae;*	Group IV (ssRNA +)	Linear	PepMV	*Solanum lycopersicum*	+	+	+^c^		
	*Tomato mosaic virus, Tobamovirus, Virgaviridae;*	Group IV (ssRNA +)	Linear	ToMV	*Solanum lycopersicum*	+	+	+^c^	KY810788	
	*Southern tomato virus, Amalgavirus, Amalgaviridae;*	Group III (dsRNA +/–)	Linear	STV	*Solanum lycopersicum*	+	+	+^b^	KY810783	
	*Columnea latent viroid, Pospiviroid, Pospiviroidae*	viroid	Circular	CLVd	*Solanum lycopersicum*	+	+	+^b^	KY810771	
IV	^*^Alfalfa mosaic virus, Alfamovirus, Bromoviridae	Group IV (ssRNA +)	Linear, segmented	AMV	*Nicotiana tabacum*	+	+	+^a^	KY810767 KY810768 KY810769	SRR5377151/SRR5377143
V	^*^Pea necrotic yellow dwarf virus, Nanovirus, Nanoviridae	Group II (ssDNA +)	Circular, segmented	PNYDV	*Pisum sativum*	+	+	+^b^	KY810774 KY810775 KY810776 KY810777 KY810778 KY810779 KY810780 KY810781	SRR5377150/SRR5377142
VI	^*^Tobacco mosaic virus, Tobamovirus, Virgaviridae	Group IV (ssRNA +)	Linear	TMV	*Nicotiana* sp.	+	+	+^b^	KY810785	SRR5377149/SRR5377141
VII	^*^Peach latent mosaic viroid, Pelamoviroid, Avsunviroidae	viroid	Circular	PLMVd	*Prunus* sp.	+	+	+^b^	KY810773	SRR5377148/SRR5377140
VIII	^*^Tomato apical stunt viroid, Pospiviroid, Pospiviroidae	viroid	Circular	TASVd	*Solanum lycopersicum*	+	+	+^b^	KY810784	SRR5377147/SRR5377139
IX	^*^Chrysanthemum stem necrosis virus, Tospovirus, Tospoviridae	Group V (ssRNA –)	Linear, segmented	CSNV	*Nicotiana benthamiana*	+	+	+^c^	MF093683 MF093684 MF093685	SRR5630913/SRR5630912

Taxonomic classification, Baltimore classification and genome organization of detected viruses are given in separate columns. Host plant information is given in the separate column. NA, not applicable; +, detected; –, not detected;

* , viruses/viroids which were known to be present in the sample before NGS analysis.

a *Confirmatory testing has been done using ELISA assay*.

b *Confirmatory testing has been done using RT-PCR assay*.

c *Confirmatory testing has been done using RT-qPCR assay*.

### Sample preparation and sequencing

Total RNA was isolated from plant samples using TRIzol reagent (Life technologies, USA) following the manufacturer's instructions. Isolated total RNA was then divided in half for comparative purposes. One half was sent to Seqmatic LLC (USA) for sRNA library preparation (TailorMix miRNA Sample Preparation Kit V2, SeqMatic LLC, USA) and sequencing. The samples were multiplexed in one lane of a HiSeq 2500 (Illumina, USA) in 1 × 50 bp mode. The remaining total RNA was further purified using an RNeasy protocol including DNase treatment following the manufacturer's protocols (RNA Cleanup protocol; RNeasy Mini Kit; Qiagen, Netherlands). Ribosomal RNA was depleted from the purified total RNA and sequencing libraries were prepared using the ScriptSeq™ Complete Kit (plant leaf) (Illumina, USA). The libraries were sequenced using MiSeq (Illumina, USA) in 2 × 300 bp (V3) mode. Number and average length of sequencing reads for every sample sequenced by both approaches are in Supplementary Table 7.

### Detection of viruses in NGS data

Reads obtained by both sequencing procedures were trimmed, filtered and further analyzed to confirm the presence of viruses and viroids. Bioinformatics pipelines used for virus detection from NGS data are detaily described in Supplementary Data 1.1. In both cases, the presence of suspected viral sequences was confirmed by mapping the reads to the complete viral genome sequences of the most similar viral isolates from the NCBI GenBank database, followed by visual inspection of individual mappings.

### Confirmatory testing

The presence of virus in each case was also confirmed by using ELISA, RT-PCR, and RT-qPCR methods (Table 1). ELISA was performed using polystyrene microtiter plates (nunc-Immuno™, Sigma-Aldrich Inc., USA) and kits containing virus specific reagents as follows, AMV: Cat No. 07001S (Loewe Biochemica GmbH, Germany), CaMV: Cat No. 07086 (Loewe Biochemica GmbH, Germany), PVY: Cat No. 1105 (Bioreba AG, Switzerland) and TYLCV: Cat. No. 1072 (Neogen Europe Ltd., UK). The assays were performed following the manufacturer's instructions. In each case a negative control corresponding to the same species as the test sample was used. The result was considered positive when the optical density (OD) A_405_ value after 2 h for a given sample was greater than 2 × the mean OD value of the corresponding negative control. For reverse transcription quantitative PCR (RT-qPCR) and reverse transcription conventional PCR (RT-PCR), total RNA was extracted from fresh or lyophilized plant material using the RNeasy Plant Mini Kit (Qiagen), following the manufacturer instructions. RT-qPCR was performed using published methods for PepMV (Gutiérrez-Aguirre et al., 2009) and for ToMV (Boben et al., 2007). Conventional RT-PCR was performed for PNYDV (Gaafar and Ziebell, 2016), STV (Sabanadzovic et al., 2009), ToCV (Dovas et al., 2002), TMV (Kumar et al., 2011), PLMVd (Loreti et al., 1999) TASVd and CLVd (Verhoeven et al., 2004). PCR primers designed specifically to confirm the presence of novel CCyV1 were as follows: CCyV1-fw (5′-GTCTCTCTTGCGTTGAGCCA-3′) and CCyV1-rev (5′-GGTTGCGGATAGCTCTTCCT-3′). All the amplicons obtained by RT-PCR were purified and sent for Sanger sequencing (GATC Biotech AG, Germany). The Sanger sequences were aligned against the genomes of detected viral species and their identity was confirmed in all of the cases.

### Construction of consensus viral/viroid genome sequences

For every identified virus/viroid the consensus viral genomes were extracted from the sRNA read mappings (see section Detection of Viruses in NGS Data) to obtain a corrected consensus genome. Validation of each corrected consensus genome was performed by mapping the *de-novo* generated contigs obtained by both NGS approaches to corresponding corrected consensus genome. Both mapping results were visually inspected for possible differences between the *de-novo* contigs and corrected consensus genome sequence. Observed conflicts were further investigated by inspecting the read mapping results. Finally, few of the observed differences were explained as polymorphisms in viral populations. In sample III, two divergent strains (80% nucleotide identity) of PepMV were detected (PepMV-EU and PepMV-CH2). In this case, the complete genome sequences of the two most similar isolates from NCBI GenBank were used in subsequent comparisons (KF718832.1 and JX866666.1), without the corrections after reads and contigs mapping as described previously.

### Comparison of sRNA and rRNA depleted totRNA inputs

For comparisons, all raw reads were trimmed and filtered in CLC Genomic Workbench 9 (Qiagen). For rRNA depleted totRNA datasets, reads shorter than 100 nucleotides were discarded. Then, reads were trimmed using quality scores, setting the limit to 0.05 (see CLC Genomics Workbench User Manual, Chapter 23, for explanation). For sRNA reads, first, adaptor trimming was performed, then reads shorter than 20 and longer than 24 nucleotides were discarded.

First, the viral fraction of the total nucleotides sequenced (from now on called percentage of virus/viroid nucleotides) in each of the datasets for each of the detected viruses was calculated by mapping the trimmed and filtered reads (of the corresponding dataset) to the consensus viral/viroid genomes generated in the previous step. Mapping parameters are listed in Supplementary Tables 1, 4.

To further compare the effectiveness of both approaches for detection and discovery of selected viruses, we then performed a normalization by subsampling the data from each sample (for both sRNA and rRNA depleted totRNA) to the same number of nucleotides. Random subsampling was performed to different subsample sizes: 1, 10, 30, and 50 million nucleotides. This was repeated ten times for each sample/size combination, yielding in total 360 datasets (9 samples × 4 subsample sizes × 10 replicates of subsampling). For those, the following analyses were implemented: (1) reads were mapped to the corresponding consensus viral/viroid genomes and the fraction of viral/viroid genome covered by reads (from now on: genome coverage (reads)) and the average depth of sequencing (number of times a nucleotide in a reference is covered by reads averaged for the complete genome) were calculated; (2) *de novo* assembly of reads was performed using CLC Genomics Workbench 9, followed by mapping the resulting contigs to the corresponding consensus viral/viroid genomes and calculation of the fraction of viral/viroid genome covered by the *de-novo* contigs (from now on: genome coverage (contigs)). Results of these comparisons are jointly shown in Figure 2 and visualized as dots connected with solid lane (representing rRNA depleted totRNA results) and triangles connected with dashed lines (representing sRNA results). The mapping and *de novo* assembly parameters are listed in Supplementary Tables 1–4.

## Results

### Sample characterization

Twelve different viruses (among those, one viral species with two divergent strains) and three viroid species were detected using NGS in the nine samples included in the analysis (Table 1). Nine were known to be present in the samples before the NGS analysis (marked with ^*^ in Table 1), whilst six virus/viroid species were detected using NGS during the study and their presence was confirmed as described in section Materials and Methods (Table 1). Both methods revealed the presence of 14 viral/viroid species whilst 1 virus (a putative novel viral species from the genus *Cytorhabdovirus*: CCyV1) could only be detected using the rRNA depleted totRNA approach. Seven samples (I, IV-IX) contained single viral/viroid infections, one sample (II) was infected with two viruses. Sample III was infected with five viruses and one viroid. All of the viruses and viroids detected and included in the study are listed in the Table 1.

### Percentage of virus/viroid reads differs for different viruses

**Figure 1:**
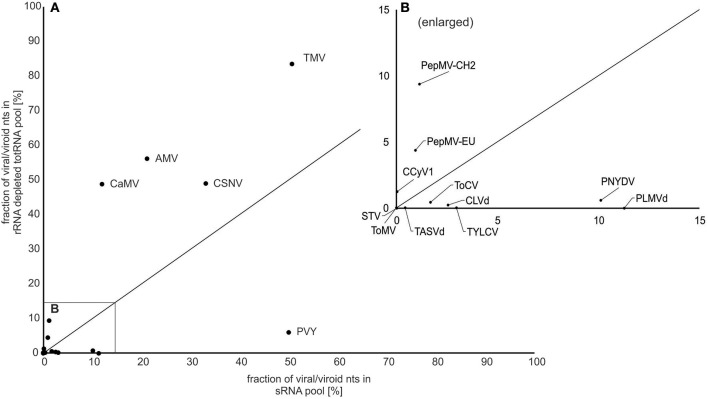
Fraction of virus/viroid nucleotides (nt) in NGS datasets for small RNA and rRNA depleted totRNA approaches. **(A)** The plots show the percentage of nucleotides (fraction of total) that mapped to the corresponding consensus viral/viroid genome for both sRNA (x-axis) and rRNA depleted totRNA (y-axis) inputs. Dots represent the value for each virus/viroid (also listed in Figure 2A), with viral/viroid species abbreviations (see Table 1) next to the dots. The diagonal solid line represents theoretical scenario in which percentage of virus/viroid nucleotides would be equivalent for both approaches; dots above the line represent cases for which fraction of virus/viroid nucleotides was higher using rRNA depleted totRNA input, dots below the line represent cases for which fraction of viruses/viroids nucleotides was higher using sRNA input. **(B)** Enlarged part of **(A)** (0–15%).

**Figure 2:**
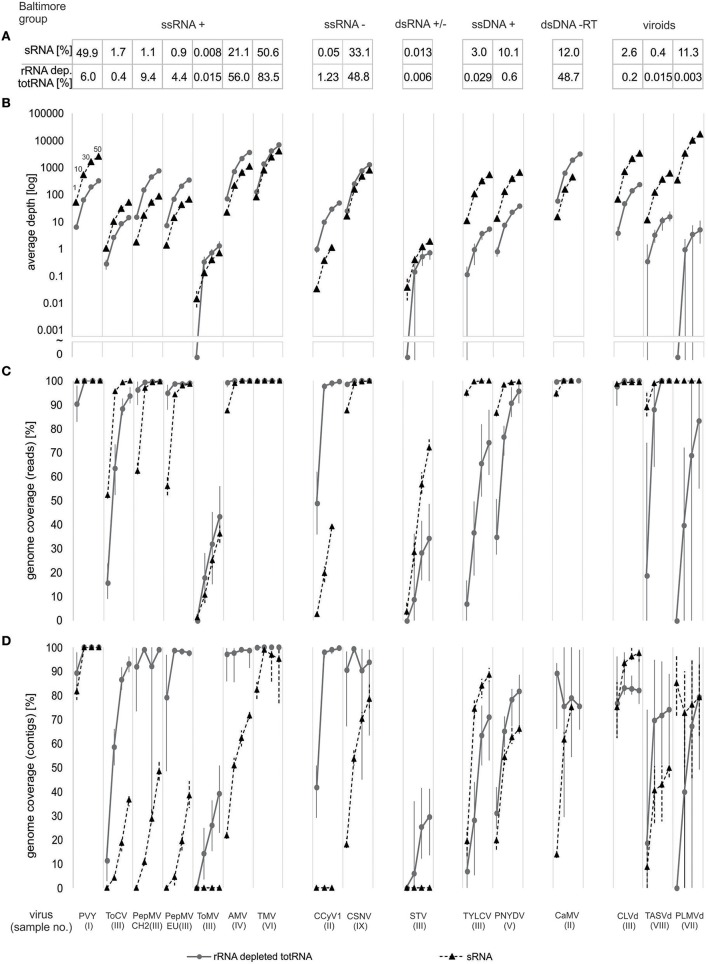
Comparison of sRNA and rRNA depleted totRNA approaches using data size-normalized subsamples. Results for each virus included in the analysis are shown along the x-axis and are grouped according to Baltimore classification **(A)** Fraction (%) of virus nucleotides in trimmed and filtered complete NGS datasets. **(B)** Average depth (number of reads covering a position in a viral genome, averaged over the complete genome sequence) at different subsample sizes. Symbol ~ indicate interruption of log scale, below, 0 values are plotted. **(C)** Fraction of viral genome (in %) covered by reads [genome coverage (reads)] at different subsample sizes. **(D)** Fraction of viral genome (in %) covered by contigs [genome coverage (contigs)] at different subsample sizes. For **(B–D)** Dots/triangles represent the mean, whereas vertical bars connect minimum and maximum results of 10 repeated analyses. Four different subsample sizes were used (1, 10, 30, and 50 million nts) and are designated in the first column, other columns follow the same logic. Triangles and dashed lines represent results for sRNA approach, dots and solid lines represent results for rRNA depleted totRNA. In some cases data points are missing, since the size of the complete dataset was smaller than the largest subsample.

First, we estimated what percentage of the total sequenced nucleotides were viral/viroid nucleotides (of the complete cleaned NGS datasets) for different viral species for each of the two approaches. The percentage of viral/viroid nucleotides was in some cases higher using sRNA input and in other cases higher using rRNA depleted totRNA input (Figure 1). Specifically, the results showed that for 6 viruses/viroids the sRNA approach generated a higher fraction of viral/viroid sequences: TASVd, ToCV, CLVd, TYLCV, PNYDV, PLMVd, and PVY (Figure 1: the viruses located below the diagonal line). For the sRNA approach, the highest percentage of viral sequences was observed for PVY (50%, Figure 2A). The rRNA depleted totRNA approach generated more viral sequences for 6 viruses: a novel *Cytorhabdovirus*, PepMV (two isolates), CaMV, AMV, CSNV and TMV (Figure 1, the viruses located above the diagonal line), with the highest viral sequences fractions for TMV (83%), AMV (56%), CSNV (48%), and CaMV (48%) (Figures 1, 2A). In two cases (STV and ToMV), the percentage of virus sequences were extremely low regardless of the RNA inputs (Figures 1, 2A).

### Comparison on normalized subsamples

To be able to compare the two approaches in a greater detail, we subsampled all of the datasets to the same number of nucleotides. Ten replicates of four different sizes of subsamples (1, 10, 30, and 50 million nucleotides) were generated for each dataset to enable an assessment of the impact of data rarefaction and data variability on the performance of tested parameters.

First, average depth was evaluated (Figure 2B). In all cases, average depth increased with the increase of subsample sizes and followed the patterns observed when comparing the fractions of viral sequences nucleotides recovered by the two approaches. Results from 10 independent replicates for each subsample size showed a low variability for PVY, ToCV, PepMV, AMV, TMV, CSNV, and CaMV. Variability between the subsamples in average depth was higher for all other viruses/viroids (Supplementary Table 5).

Secondly, we investigated how effectively the reads cover the genomes of different viruses by calculating the fraction of the genome covered by reads [genome coverage (reads)] (Figure 2C). Results of the analysis showed low variability between replicates of subsamples, except when mapping rRNA depleted totRNA reads to ToMV, STV, TYLCV, TASVd, and PLMVd where variation was very high (Supplementary Table 5, Figure 2C). In all cases, as expected, better genome coverage was achieved with the increasing subsample sizes. For the sRNA approach, complete genomes (100%) were covered for majority of the viruses/viroids at subsample size of 30 million nucleotides. The exceptions were ToMV, STV and the putative novel *Cytorhabdovirus*. For those, even at 50 million nucleotides, genome coverage was 70% or less.

For the rRNA depleted totRNA approach, for half of the viruses (PVY, PepMV, AMV, TMV, novel CCyV1, CSNV, CaMV, CLVd, and TASVd) complete genomes were covered at 10 million nucleotides. However, for some viruses/viroids (ToCV, TYLCV, PNYDV, and PLMVd) relatively low genome coverage was achieved at smaller subsample sizes (1 and 10 million nts) and even at the largest subsample size (50 million nts) the coverage did not reach 100% (Figure 2C). The genomes of ToMV and STV, for which very low numbers of reads were recovered (Figures 1, 2A), were poorly covered even at high subsampling depths, for example, even with 50 million nucleotides, coverage remained below 50% (Figure 2C).

Reads from normalized datasets were *de novo* assembled into contigs, which were then mapped to the corresponding consensus viral genomes in order to calculate the fraction of the viral genomes covered by the *de novo* assembled contigs [genome coverage (contigs)] (Figure 2D). The analysis of subsample replicates showed in general lower variability for sRNA datasets than rRNA depleted totRNA datasets (Supplementary Table 5). For the majority of the viruses, the coverage by contigs increased with subsample size, however, conversely, in several cases, it dropped at larger subsample sizes, i.e., TMV and PLMVd for sRNA and PepMV, CSNV, CaMV and CLVd for rRNA depleted totRNA approach (Figure 2D). Contigs, assembled *de novo* from rRNA depleted totRNA datasets covered higher fractions of viral genomes for almost all viruses at all subsample sizes (coverage reached 95% at 10 million nts for majority of viruses), in comparison to sRNA derived contigs (95% coverage at 10 million nts was achieved only for PVY, TMV, and CLVd). Two exceptions to this observation were TYLCV and CLVd, for which sRNA derived *de novo* contigs cover higher genome fraction than rRNA depleted totRNA contigs, for all subsample sizes.

The comparison of the *de novo* assemblies for STV and ToMV revealed that when very low numbers of viral reads are recovered, the rRNA depleted totRNA approach is more effective, since in the case of the sRNA approach, no corresponding viral contigs were generated (Figure 2D). A similar scenario was observed also for the putative novel *Cytrohabovirus*, where very low recovery of viral reads in the sRNA dataset resulted in no assembled contigs corresponding to this virus (Figure 2D).

## Discussion

In this study we compared the effectiveness of two NGS approaches that have been widely adopted for plant virus detection: sRNA deep sequencing and deep sequencing of rRNA depleted totRNA. When comparing the amount of virus/viroid reads recovered by one or the other approach, we observed different results for different viruses/viroids: in some cases, more viral/viroid nucleotides were recovered using sRNA and in other by rRNA depleted totRNA sequencing.

Detailed inspection of the results of the read mapping suggested higher recovery of virus reads for ssDNA viruses and viroids when using sRNA approach than when using rRNA depleted totRNA approach. For viroids, this could be the consequence of induced RNA silencing (Itaya et al., 2001; Papaefthimiou et al., 2001; Martínez de Alba et al., 2002) and, at the same time, the absence of the messenger RNA production, because, in the case of viroids, “long” RNAs are generated solely for the purpose of replication. Similarly, in the case of viruses with a circular ssDNA genome organization, a smaller fraction of viral nucleotides was recovered using rRNA depleted totRNA. In contrast with viruses with RNA genomes, for ssDNA viruses, RNA molecules are generated only during the transcription step, as messenger RNAs, which could be the reason for the lower recovery of viral nucleotides in this pool. Moreover, small RNAs could be amplified by the action of RNA-dependent RNA polymerase 6 (Borges and Martienssen, 2015) during the production of secondary sRNAs. The exception among the DNA viruses in this study was CaMV (DNA-RT), for which a higher fraction of virus nucleotides was recovered by sequencing rRNA depleted totRNA. The CaMV dsDNA genome is replicated through an RNA intermediary, in addition to producing messenger RNAs through transcription (Hull, 2014), which could explain a larger proportion of viral nucleotides in this pool.

All linear viruses in our infected plant samples had a ssRNA genome organization and synthesize different types of RNA throughout their replication cycle. For most of these viruses, sequencing rRNA depleted totRNA resulted in a larger proportion of reads mapping to the viral genomes (Figure 1) compared with sRNA. However, a few exceptions were observed, PVY being the most notable with many more viral reads being present in the sRNA dataset. The high abundance of virus derived sRNA has already been reported for PVY (Kutnjak et al., 2015) and other potyviruses (Kreuze et al., 2009) even though they encode strong RNA silencing suppressors (Yelina et al., 2002; Ivanov et al., 2016).

In general, when read mapping was performed, 10 million nucleotides was sufficient to cover complete viral genomes using any of the two approaches (Figure 2C). However, in some cases (STV and ToMV in sample III) very low numbers of viral reads were recovered (by both approaches), which negatively affected all the evaluated parameters. For those two cases, the percentage of virus reads (for both approaches) was lower than 0.1%, and the average read depth remained lower than 10×, and none of the viral genomes were completely covered by the reads even at the highest subsample size (50 million) (Figure 2C).

When comparing *de novo* assembly of sequencing reads, the rRNA depleted totRNA approach was generally more efficient than sRNA approach; this was demonstrated in higher proportion of viral genomes covered by *de novo* generated contigs from rRNA depleted totRNA datasets. The contigs assembled from rRNA depleted totRNA data covered at least a fraction of the consensus genome even in cases where the percentage of virus/viroid reads was lower than 0.1% and average depth lower than 10 (i.e., ToMV and STV) (Figure 2D). In those cases, no viral contigs were assembled using sRNA datasets, probably due to a combination of low amount and small sizes of viral reads. Poorer coverage of viral genomes by sRNA derived *de novo* contigs is likely related to the more difficult assembly of very short sRNA reads into longer contigs, which has been observed previously (Kutnjak et al., 2015; Visser et al., 2016).

In some cases (PepMV, TMV, CSNV CaMV, CLVd, and PLMVd) smaller genome fractions are covered by contigs, when larger data sets are used for the assembly (corresponding to average depths > 100). This has been observed previously and is an artifact of the assembly algorithms (see CLC Analyses-related questions, 2017), which are not optimized for very high sequencing depths. After mapping reads or contigs to evaluate average depth and genome coverage (reads/contigs) we observed also the trend in generating higher or lower variability within 10 repeats. Unrepeatable random subsampling occurred when analysing smaller datasets and/or lower viral/viroid nucleotide proportion within the datasets, since all samples with this two features had greater variability.

The study has highlighted some points of difference between the compared approaches that may help to inform the choice of approach based on the purpose of the sequencing. This could be (i) screening against a list of known target organisms (e.g., at the import/export) and (ii) identification of the (possibly yet unknown) causal agent of the disease. Considering (i) screening against a list of known targets, this would be most cost effectively achieved using a method that maximizes the amount of viral sequences compared with host sequences. This study showed (Figures 1, 2A) that the performance of the two compared approaches is very virus dependent. Broadly, sRNA performed better for circular ssDNA viruses and viroids, whilst rRNA depleted total RNA performed better for most of the tested linear RNA viruses with a notable exception (PVY). If considering (ii) sequencing for novel virus discovery, long contigs would provide the greatest chance of detecting very dissimilar sequences by comparing predicted amino-acid sequence from virus ORFs (e.g., with the use of BLASTx analysis or hidden Markov model based protein domain searches). The data shows that rRNA depleted total RNA generated longer contigs (which covered greater fractions of viral genomes) for most of the investigated viruses (Figure 2D). As the most prominent example, an important difference between the compared approaches was observed on a case of a previously un-described *Cytorhabdovirus*, which was identified from the rRNA depleted total RNA following *de novo* assembly and BLASTx analysis, whilst the virus reads could only be found in the sRNA sequence data *post-hoc* (de novo assembly of sRNA reads did not generate any matching contigs).

The results of the comparison between the two NGS approaches highlight some trends that may guide diagnostic laboratories in the selection of a method appropriate for a specific application. However, whichever method is selected it is important to be aware of the limitations, some of which are detailed in this study, and follow up putative identification using an appropriate method. The recently published framework for handling novel plant viruses detected using NGS provides guidelines for achieving this (Massart et al., 2017).

In order to examine the potential costs of each method on commonly used Illumina sequencing platforms (HiSeq/sRNA and MiSeq/rRNA depleted totRNA) staff time used and reagent costs (in GBP) were calculated using list prices (Illumina) obtained on 1st March 2017. In general, both approaches generate more than sufficient amount of data than required to identify all of the viruses if mapping is used (50 million nts; Figure 2). HiSeq/sRNA sample will cost £138 and MiSeq/rRNA depleted totRNA sample will cost £159 if 24 samples (reasonable diagnostic throughput) are run per lane / flow cell, which is comparable price for output of 24 samples. Detail information about calculations is described in Supplementary data 1.2 and in Supplementary Table 6.

The outcomes presented in this study showed that all included known viruses/viroids could be identified by both NGS approaches. Both approaches successfully identified also two divergent strains of PepMV, which was, despite short fragments of sRNA already shown previously (Kutnjak et al., 2014). However, a putative novel *Cytorhabdovirus* was only detected by analysing the data generated from ribosomal RNA depleted total RNA. Additionally, the results revealed the strength of NGS technology for the simultaneous detection and identification of several different known/unknown plant viruses from a different sample material, with a different amount of viral/viroid nucleotides and in a different host plants. Similar conclusions were derived from studies using other virus enrichment approaches on single or few viral species (Adams et al., 2009; Al Rwahnih et al., 2009; Kreuze et al., 2009; Kutnjak et al., 2014; Visser et al., 2016), e.g., both, sequencing of virion-associatad nucleic acids and sRNAs enabled a discovery of a new virus, previously overlooked by other detection techniques (Candresse et al., 2014). Our study further indicates the advantages of NGS in such cases and strengthens its use as a tool in plant virus/viroid diagnostics.

## Author contributions

MR, DK, and NB conceived the idea, AP, MR, DK, and NB designed the experiments. AF provided samples. AP performed laboratory part of the experiment and analyzed the data with the assistance of IA and DK. AP wrote the draft of the manuscript. All authors significantly contributed with reviewing and editing the manuscript.

### Conflict of interest statement

The authors declare that the research was conducted in the absence of any commercial or financial relationships that could be construed as a potential conflict of interest.
