# Supplementary material for: Next Generation Sequencing for Detection and Discovery of Plant Viruses and Viroids: Comparison of Two Approaches
Source: Front Microbiol. 2017 Oct 13;8:1998. doi: 10.3389/fmicb.2017.01998 (PMC5645528; doi:10.3389/fmicb.2017.01998)
Supplement: Supplementary file 1 [file DataSheet1.DOCX]

Supplementary Material

**Next generation sequencing for detection and discovery of plant viruses and viroids: comparison of two approaches**

**Anja Pecman^1,2*^, Denis Kutnjak^1*^, Ion Gutierrez Aguirre^1^, Ian Adams^3^, Adrian Fox^3^, Neil Boonham^3,4^, Maja Ravnikar^1^**

*** Correspondence:**Corresponding Author
anja.pecman@nib.si, denis.kutnjak@nib.si

# Supplementary Data

## Virus/viroid detection

For sRNA, analysis was performed using CLC Genomics Workbench 9: first, adapters were trimmed from the reads, then reads were filtered by length (only reads 20-24 nucleotides long, corresponding to the size of small interfering RNAs, were retained). Trimmed and size-selected reads were first mapped (parameters given in Supp. Table 1) to the NCBI Viral RefSeq database, containing representatives of all viral genomes with completely sequenced genomes. Results of the mapping were manually inspected. In parallel de novo assembly of trimmed and size-selected reads was performed (parameters given in Supp. Table 2). Assembled contigs were compared for similarity against all viral sequences deposited in the NCBI GenBank nt database using BLASTn and BLASTx.

Ribosomal RNA depleted totRNA sequencing reads were trimmed for length and quality; reads shorter that 100 nucleotides and with a Q (Pherd quality) score below 20 were discarded using Sickle (Joshi & Fass, 2011). De-novo assembly of trimmed and filtered reads was carried out using Trinity DNA assembler (Grabherr et al. 2013). Assembled contigs were compared for similarity against all viral sequences deposited in the NCBI GenBank nt database using BLASTn and BLASTx algorithms and the Blast+ tool (Camacho et al., 2009). The results were visualized using Megan DNA 5 (Huson, et. al. 2007).

In both cases the presence of suspected viral sequences was confirmed by mapping the reads to the complete viral genome sequences of the most similar viral isolates from the NCBI GenBank database, followed by visual inspection of individual mappings.

## Cost calculation for sequencing

In order to examine the potential costs of both methods prices for reagents for the HiSeq /sRNA and MiSeq/rRNA depleted totRNA methods were calculated using list prices from the Illumina website. The prices were obtained in pounds on 1st March 2017. The cost of consumables for a 50 bp single lane on a HiSeq 3000 was approximately £826 and for a 2x300 bp run on a MiSeq £1253. Staff time also needs to be accounted for. It takes approximately 60 min to run a MiSeq once the library is prepared and although the HiSeq takes longer, 8 lanes are prepared simultaneously so an estimate of 60 min per lane is not unreasonable. In both cases that means 2.5 minutes per sample. The Illumina TruSeq small RNA kit costs approximately £2005 for 24 reactions and according to the manual takes 4 hrs hands on time. Prior to this kit the small RNA requires gel purification. We estimate this as costing £20 per sample and taking 4 hr for 24 samples. This gives a final cost of £86 per sample and 20 minutes of staff time. The ScriptSeq complete kit for ribosome depletion and library preparation costs approximately £2558 for 24 reactions and takes 8 hrs for 24 samples. This gives a final cost of £106 per sample and 20 mins of staff time. In total if 24 samples (reasonable diagnostic throughput) are run per lane / flow cell, a HiSeq sample will cost £138 and 22.5 min of staff time to deliver 230 million raw (or 105 million adapter and length trimmed) nts of sRNA data and a MiSeq sample will cost £159 and 22.5 min of staff time to deliver 504 million raw (or 458 million adapter and length trimmed) nts of rRNA depleted totRNA data (Supp. Table 6). Despite the difference in nts numbers, both methods generate more than sufficient amount of data as required to identify all of the viruses if mapping is used (50 million nts; Figure 2). In order to generate 50 million nts per sample this also equates to 48 samples on a HiSeq lane (52,708,333 nts per sample) or 200 samples on a MiSeq flow cell (55,055,000 nts per sample) (Supplementary Table 6).

# Supplementary Figures and Tables

## Supplementary Tables

Supplementary Table 1. Parameters used in CLC Genomic Workbench 9 for mapping reads to consensus viral/viroid genomes

| **Parameter** | **Parameter value** |
| --- | --- |
| References | consensus viral/viroid genomes |
| Masking mode | No masking |
| Masking track |  |
| Match score | 1 |
| Mismatch cost | 1 |
| Cost of insertions and deletions | Linear gap cost |
| Insertion cost | 2 |
| Deletion cost | 2 |
| Insertion open cost | 6 |
| Insertion extend cost | 1 |
| Deletion open cost | 6 |
| Deletion extend cost | 1 |
| Length fraction | * |
| Similarity fraction | ** |
| Global alignment | false |
| Color space alignment | false |
| Color error cost | 3 |
| Auto-detect paired distances | true |
| Non-specific match handling | Map randomly |

Supplementary Table 2. Parameters used in CLC Genomic Workbench 9 for *de novo* assembly

| **Parameter** | **Parameter value** |
| --- | --- |
| Mapping mode | Create simple contig sequences (fast) |
| Update contigs | true |
| Mismatch cost | 2 |
| Insertion cost | 3 |
| Deletion cost | 3 |
| Colorspace error cost | 3 |
| Length fraction | 0.5 |
| Similarity fraction | 0.8 |
| Colorspace alignment | true |
| Alignment mode | local |
| Match mode | random |
| Create list of un-mapped reads | false |
| Automatic bubble size | true |
| Bubble size | 50 |
| Automatic word size | true |
| Word size | 20 |
| Minimum contig length | 50 |
| Guidance only reads |  |
| Perform scaffolding | true |
| Auto-detect paired distances | true |
| Create report | true |

Supplementary Table 3. Parameters used in CLC Genomic Workbench 9 for mapping *de novo* assembled contigs to consensus viral/viroid genomes

| **Parameter** | **Parameter value** |
| --- | --- |
| References | consensus viral/viroid genomes |
| Masking mode | No masking |
| Masking track |  |
| Match score | 1 |
| Mismatch cost | 1 |
| Cost of insertions and deletions | Linear gap cost |
| Insertion cost | 2 |
| Deletion cost | 2 |
| Insertion open cost | 6 |
| Insertion extend cost | 1 |
| Deletion open cost | 6 |
| Deletion extend cost | 1 |
| Length fraction | # |
| Similarity fraction | ## |
| Global alignment | false |
| Color space alignment | false |
| Color error cost | 3 |
| Auto-detect paired distances | true |
| Non-specific match handling | Map randomly |

Supplementary Table 4. Adjusted^+^ parameters used in CLC Genomic Workbench 9 for mapping reads and *de novo* assembled contigs to consensus viral/viroid genomes. Linear viruses are: PVY, ToCV, PepMV, ToMV, AMV, TMV, novel CCyV1, CSNV and STV; while circular viruses/viroids are: TYLCV, PNYDV, CaMV, CLVd, TASVd and PLMVd. For signs * and ** see the Supplementary Table 1 and for signs # and ## see the Supplementary Table 3.

| Virus | **parameters sRNA reads */**** | **parameters sRNA contigs #/##** | **parameters totRNA reads */**** | **parameters totRNA contigs #/##** |
| --- | --- | --- | --- | --- |
| Linear viruses | 0.95/0.95 | 0.95/0.90 | 0.95/0.95 | 0.95/0.90 |
| Circular viruses/viriods | 0.95/0.95 | 0.50/0.90 | 0.50/0.95 | 0.50/0.90 |

^+^Small circular genome organization could influence mapping efficiency (Visser, 2016), especially in the case of longer reads, so parameters for mapping contigs from both datasets and reads from rRNA depleted totRNA to all circular viruses/viroids were adjusted. The length fraction parameter (the minimum percentage of the total alignment length that must match the consensus genome at the selected similarity fraction) was set to 0.95 (95 %) for all viruses, except circular viruses/viroids, for which it was adjusted to 0.5 (50 %).

Supplementary Table 5. Coefficient of variation (CV) for all viruses/viroids at all data sizes (1, 10, 30 and 50 millions of nucleotides) for both approaches (rRNA depleted totRNA and sRNA). The calculation has been done for average depth, consensus length (reads) and consensus length (de novo contigs).

|  |  |  | CV (rRNA depleted totRNA) [%] | | | CV (sRNA) [%] | | |
| --- | --- | --- | --- | --- | --- | --- | --- | --- |
| Sample no. | virus | DATASET SIZE [million nts] | (average depth) | (consesnsus length (reads)) | (consesnsus length (*de novo* contigs)) | (average depth) | (consesnsus length (reads)) | (consesnsus length (*de novo* contigs)) |
| I | PVY | 1 | 11.55 | 6.04 | 6.61 | 0.39 | 0.02 | 2.17 |
|  |  | 10 | 2.51 | 0.02 | 0.02 | 0.18 | 0.00 | 0.22 |
|  |  | 30 | 1.33 | 0.01 | 0.01 | 0.06 | 0.00 | 0.06 |
|  |  | 50 | 1.41 | 0.00 | 0.01 | 0.04 | 0.00 | 0.19 |
| III | ToCV | 1 | 32.96 | 28.97 | 40.86 | 2.17 | 2.09 | 0.00 |
|  |  | 10 | 10.27 | 8.31 | 6.84 | 1.39 | 0.52 | 16.00 |
|  |  | 30 | 3.95 | 3.09 | 3.48 | 0.70 | 0.14 | 10.09 |
|  |  | 50 | 3.55 | 2.44 | 2.35 | 0.27 | 0.11 | 4.11 |
| III | PepMV-CH2 | 1 | 7.71 | 2.84 | 9.70 | 4.29 | 3.20 | 316.23 |
|  |  | 10 | 2.58 | 0.50 | 0.45 | 1.02 | 0.43 | 10.19 |
|  |  | 30 | 1.38 | 0.41 | 23.70 | 0.89 | 0.24 | 6.68 |
|  |  | 50 | 1.06 | 0.21 | 0.52 | 0.63 | 0.18 | 6.09 |
| III | PepMV-EU | 1 | 10.07 | 3.50 | 20.17 | 4.87 | 4.06 | 0.00 |
|  |  | 10 | 3.75 | 0.12 | 0.18 | 1.29 | 0.51 | 37.02 |
|  |  | 30 | 1.99 | 0.18 | 0.69 | 0.55 | 0.40 | 12.72 |
|  |  | 50 | 0.95 | 0.08 | 0.51 | 0.71 | 0.21 | 8.57 |
| III | ToMV | 1 | 0.00 | 0.00 | 0.00 | 43.83 | 45.68 | 0.00 |
|  |  | 10 | 41.23 | 35.95 | 43.93 | 18.34 | 16.44 | 0.00 |
|  |  | 30 | 29.05 | 31.98 | 26.67 | 7.10 | 5.68 | 0.00 |
|  |  | 50 | 21.83 | 17.07 | 21.61 | 6.68 | 6.67 | 0.00 |
| IV | AMV | 1 | 1.57 | 0.86 | 4.33 | 1.24 | 0.77 | 4.79 |
|  |  | 10 | 0.55 | 0.07 | 4.40 | 0.29 | 0.35 | 3.55 |
|  |  | 30 | 0.23 | 0.04 | 0.60 | 0.16 | 0.12 | 3.06 |
|  |  | 50 | 0.31 | 0.02 | 2.61 | 0.16 | 0.08 | 1.84 |
| VI | TMV | 1 | 1.04 | 0.09 | 0.17 | 0.55 | 0.16 | 2.76 |
|  |  | 10 | 0.41 | 0.00 | 0.10 | 0.10 | 0.00 | 0.66 |
|  |  | 30 | 0.21 | 0.00 | 0.04 | 0.10 | 0.00 | 5.28 |
|  |  | 50 | 0.15 | 0.01 | 0.17 | 0.03 | 0.00 | 8.79 |
| II | CCyV1 | 1 | 19.94 | 17.60 | 18.01 | 14.86 | 16.86 | 0.00 |
|  |  | 10 | 4.92 | 0.99 | 1.05 | 8.61 | 9.96 | 0.00 |
|  |  | 30 | 2.01 | 0.77 | 0.96 | 0.81 | 0.91 | 0.00 |
|  |  | 50 | 2.99 | 0.09 | 0.23 | / | / | / |
| IX | CSNV | 1 | 3.14 | 0.62 | 11.00 | 1.14 | 1.04 | 6.66 |
|  |  | 10 | 0.61 | 0.06 | 0.61 | 0.22 | 0.21 | 3.71 |
|  |  | 30 | 0.28 | 0.02 | 19.34 | 0.13 | 0.17 | 2.46 |
|  |  | 50 | 0.22 | 0.00 | 11.56 | 0.08 | 0.09 | 3.95 |
| III | STV | 1 | 0.00 | 0.00 | 0.00 | 59.32 | 57.04 | 0.00 |
|  |  | 10 | 118.35 | 128.54 | 189.17 | 15.62 | 11.06 | 0.00 |
|  |  | 30 | 44.79 | 32.12 | 38.53 | 4.87 | 5.24 | 0.00 |
|  |  | 50 | 24.17 | 25.08 | 31.75 | 3.74 | 2.37 | 0.00 |
| III | TYLCV | 1 | 88.81 | 94.56 | 94.56 | 3.22 | 1.21 | 20.92 |
|  |  | 10 | 43.79 | 25.54 | 42.76 | 0.73 | 0.36 | 3.18 |
|  |  | 30 | 23.74 | 16.56 | 14.30 | 0.48 | 0.14 | 2.87 |
|  |  | 50 | 12.77 | 11.56 | 16.02 | 0.36 | 0.20 | 2.00 |
| V | PNYDV | 1 | 26.77 | 20.20 | 18.31 | 1.28 | 1.17 | 9.44 |
|  |  | 10 | 12.55 | 4.89 | 6.74 | 0.48 | 0.18 | 3.04 |
|  |  | 30 | 7.46 | 3.69 | 5.82 | 0.17 | 0.14 | 3.90 |
|  |  | 50 | 5.28 | 2.87 | 8.86 | 0.06 | 0.05 | 1.76 |
| II | CaMV | 1 | 2.75 | 0.59 | 9.28 | 1.52 | 1.10 | 8.91 |
|  |  | 10 | 1.09 | 0.00 | 39.66 | 0.31 | 0.03 | 2.25 |
|  |  | 30 | 0.58 | 0.00 | 24.20 | 0.06 | 0.00 | 1.57 |
|  |  | 50 | 0.20 | 0.00 | 14.02 | / | / | / |
| III | CLVd | 1 | 37.13 | 4.45 | 14.12 | 3.27 | 0.60 | 11.60 |
|  |  | 10 | 7.87 | 0.00 | 6.91 | 0.90 | 0.09 | 2.70 |
|  |  | 30 | 11.89 | 0.00 | 3.73 | 0.42 | 0.13 | 2.43 |
|  |  | 50 | 3.80 | 0.00 | 7.52 | 0.34 | 0.09 | 2.36 |
| VIII | TASVd | 1 | 156.18 | 160.52 | 160.52 | 5.96 | 4.12 | 108.66 |
|  |  | 10 | 36.69 | 15.74 | 29.90 | 1.60 | 1.17 | 22.35 |
|  |  | 30 | 28.27 | 0.00 | 19.85 | 0.62 | 0.26 | 22.49 |
|  |  | 50 | 28.99 | 0.00 | 18.77 | 0.57 | 0.00 | 3.51 |
| VII | PLMVd | 1 | 0.00 | 0.00 | 0.00 | 1.84 | 0.00 | 12.46 |
|  |  | 10 | 92.43 | 86.79 | 86.79 | 0.35 | 0.00 | 18.51 |
|  |  | 30 | 63.78 | 40.15 | 37.85 | 0.16 | 0.00 | 21.68 |
|  |  | 50 | 57.76 | 19.02 | 22.92 | 0.13 | 0.00 | 14.05 |

Supplementary Table 6. Detail information about sequencing (HiSeq/sRNA and MiSeq/totRNA) costs, time required and output obtained.

| Sample number = 24 | HiSeq/sRNA | MiSeq/totRNA |
| --- | --- | --- |
| COSTING | | |
| Illumina run kit (FC 410-1001)/(V3 600) | £1,801.00 | £1,253 |
| Illumina cluster kit (FPE-410-1001) | £4,813.00 | / |
| cost per lane/run | £826.75 | £1,253 |
| **sequencing cost per sample** | **£34.45** | **£52.21** |
| Illumina TruSeq small RNA library preparation kit + gel/sample | £103.54 | / |
| Illumina ScriptSeq complete kit (plant leaf)/sample | / | £106.58 |
| **total cost per sample** | **£137.99** | **£158.79** |
| TIME | | |
| staff time (minute) /sample | 22.5 | 22.5 |
| OUTPUT | | |
| reads/sample | 10,416,667 | 1,041,667 |
| nucleotides/sample | 229,166,667 | 504,166,667 |
| trimmed nucleotides/sample | 105,416,667 | 458,791,667 |

Supplementary Table 7. Number of raw reads, trimmed reads and average length of trimmed reads for every sample sequenced by both approaches.

| Sample no. | sRNA reads | | | rRNA depleted totRNA reads | | |
| --- | --- | --- | --- | --- | --- | --- |
|  | Reads count before trimming | Reads count after trimming described in manuscript section 2.6 | Average reads length after trimming | Reads count before trimming | Reads count after trimming described in manuscript section 2.6 | Average reads length after trimming |
| I | 10,718,277 | 4,072,350 | 21 | 3,701,286 | 2,853,754 | 210 |
| II | 9,853,075 | 1,524,799 | 22 | 1,588,384 | 1,482,444 | 257 |
| III | 10,877,206 | 7,016,674 | 23 | 1,256,386 | 1,151,128 | 248 |
| IV | 10,853,294 | 7,230,019 | 23 | 3,307,272 | 3,208,198 | 250 |
| V | 14,117,464 | 2,712,946 | 22 | 2,198,024 | 1,980,650 | 233 |
| VI | 10,215,129 | 5,237,655 | 22 | 3,721,544 | 3,463,112 | 241 |
| VII | 16,524,330 | 6,700,322 | 22 | 1,948,330 | 1,816,834 | 239 |
| VIII | 14,622,679 | 4,604,339 | 23 | 3,734,788 | 3,543,264 | 245 |
| IX | 11,958,036 | 8,358,087 | 22 | 2,343,928 | 1,623,012 | 183 |

# References

Camacho, C., Coulouris, G., Avagyan, V., Ma, N., Papadopoulos, J., Bealer, K., & Madden, T. L. (2009). BLAST plus: architecture and applications. *BMC Bioinformatics*, *10*(421), 1. https://doi.org/Artn 421\nDoi 10.1186/1471-2105-10-421

Grabherr, M. G. ., Brian J. Haas, Moran Yassour Joshua Z. Levin, Dawn A. Thompson, Ido Amit, Xian Adiconis, Lin Fan, Raktima Raychowdhury, Qiandong Zeng, Zehua Chen, Evan Mauceli, Nir Hacohen, Andreas Gnirke, Nicholas Rhind, Federica di Palma, Bruce W., N., & Friedman, and A. R. (2013). Trinity: reconstructing a full-length transcriptome without a genome from RNA-Seq data. *Nature Biotechnology*, *29*(7), 644–652. https://doi.org/10.1038/nbt.1883.Trinity

Huson, D., Auch, A., Qi, J., & Schuster, S. (2007). MEGAN analysis of metagenome data. *Gennome Res.*, *17*, 377–386. https://doi.org/10.1101/gr.5969107.

Joshi, N. A., & Fass, J. N. (2011). *Sickle: A sliding-window, adaptive, quality-based trimming tool for FastQ files*. Retrieved from http://github.com/najoshi/sickle

Visser, M., Bester, R., Burger, J. T., & Maree, H. J. (2016). Next-generation sequencing for virus detection: covering all the bases. *Virology Journal*, *13*(1), 85. https://doi.org/10.1186/s12985-016-0539-x
